# Hyaluronic Acid-Based Nanomaterials for Cancer Therapy

**DOI:** 10.3390/polym10101133

**Published:** 2018-10-12

**Authors:** Jin Hong Kim, Myeong Ju Moon, Dong Yi Kim, Suk Hee Heo, Yong Yeon Jeong

**Affiliations:** 1 Department of Surgery, Chonnam National University Medical School, Gwangju 61469, Korea; abyss1015@naver.com (J.H.K.); dockim@jnu.ac.kr (D.Y.K.); 2 Department of Radiology, Chonnam National University Medical School, Chonnam National University Hwasun Hospital, Hwasun 58128, Korea; mjmoon2398@gmail.com

**Keywords:** hyaluronic acid (HA), nanoparticles (NPs), cancer, target therapy, chemotherapy, gene deliver, immunotherapy, combination cancer therapy

## Abstract

Hyaluronic acid (HA) is a nonsulfated glycosaminoglycan and a major component of the extracellular matrix. HA is overexpressed by numerous tumor cells, especially tumor-initiating cells. HA-based nanomaterials play in importance role in drug delivery systems. HA is used in various types of nanomaterials including micelle, polymersome, hydrogel, and inorganic nanoparticle formulations. Many experiments show that HA-based nanomaterials can serve as a platform for targeted chemotherapy, gene therapy, immunotherapy, and combination therapy with good potential for future biomedical applications in cancer treatment.

## 1. Introduction

Successful cancer treatment is a major element of recent medical practice. Systemic toxicity and the lack of tumor selectivity have hindered many new chemotherapeutic molecules in reaching clinical translation. The way to decrease selective harm in cancer treatment is by actively targeting tumor cells by applicating the anatomical, pathophysiological, and microenvironmental differences between malignant areas and normal bodily tissues [1].

Hyaluronic acid (HA) is a linear mucopolysaccharide consisted of alternately repeated *N*-acetylglucosamine and glucuronic di-saccharide, and it makes up a major part of extracellular matrix. HA has hydroxyl and carboxylic groups, as well as an *N*-acetyl group, which can be used for further chemical modifications. A large variety of cells, such as fibroblasts, synthesize HA [2]. HA shows superior physiochemical natures, such as a high water-binding capacity, nontoxicity, biodegradability, cytocompatibility, and nonimmunogenicity [3]. Due to these biological abilities of HA, there is a great interest in the development of HA-based nanomaterials for diverse biomedical applications, including drug delivery systems (DDS) and molecular imaging.

Many cancer cells are known to overexpresses HA-biding receptors, such as CD44, LYVE-1 receptors, and RHAMM [4]. HA is degraded to low molecular weight components by hyaluronidase after being taken up by cancer cells through CD44 receptor-mediated endocytosis [5]. CD44, a glycoprotein ubiquitous throughout the body, carries great potential for fulfilling the promise of active targeting. Overexpressed CD44 receptor showed in various cancer cells, including those of colon, ovarian, breast, and squamous carcinoma [6].

HA has been often modified with a drug carrier to improve drug delivery to CD44-overexpressing cancer cells to effectively suppress cancer growth due to its ability to specifically target CD44 [4]. HA is widely used in nanoparticle (NP) formations in experimental studies. NP formulations have attracted scientific researchers’ attention because of their ability to take advantage of the enhanced permeability and retention effect for the tumor areas [2,7]. By conjugating an active moiety, such as HA, to the surface of NPs, the cancer cell selectivity can be enhanced through active targeting [8].

In this review, we focus on the recent methodological developments in HA-based nanomaterials to treat tumors. In addition, this review demonstrates nanodelivery systems using HA for encapsulating and targeting active molecules, as well as alternative approaches for targeting CD44 in cancer therapy.

## 2. HA-Based Nanomaterials

**Figure 1:**
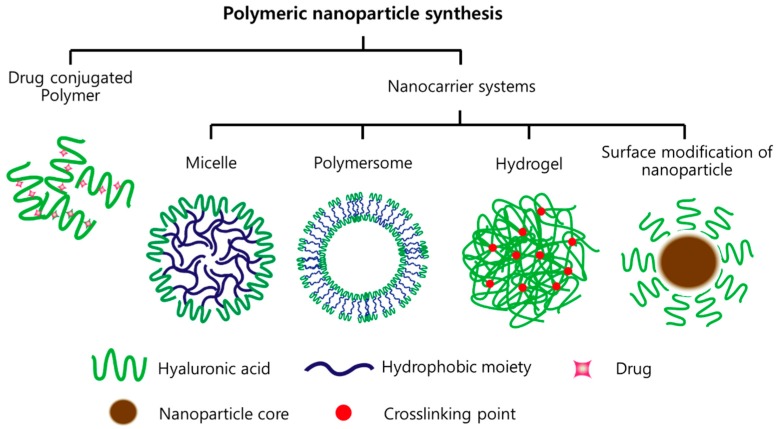
Formulations of hyaluronic acid (HA)-based nanomaterials.

HA has several functional groups used for various conjugations and modification. These properties make HA a major component of multifunctional NPs to deliver synergistic cancer therapies [9]. Several approaches for producing HA NP formulations have developed to take advantage of the targeting properties of HA [2,3,4,5,10,11]. Reported HA-based nanomaterials for cancer treatment include polymeric drug-conjugated HA and nanomaterials, such as micelles, polymersome, hydrogels, and inorganic NP systems, as shown in Figure 1. HA nanomaterials have several merits that are low to no immunogenicity, non-inflammatory reactions, biodegradability, biocompatibility, and bioavailability [12].

### 2.1. Drug-Conjugated HA

HA is a large hydrophilic biopolymer of repeating disaccharide units, and it can be directly conjugated to drugs. Direct conjugation of HA to anticancer drugs generates new compounds with promising antitumor effects [13,14,15,16,17,18]. Such simple yet effective NP formulations can be used to improve treatment efficacy because HA-targeted receptors (CD44) are overexpressed in many cancers. Aside from this targeting ability, drug-conjugated HA provides merits in terms of increasing circulation time, drug stability, solubility, and cancer-targeting ability [12,19]. Once internalized, drug-conjugated HA is hydrolyzed by intracellular enzymes and releases the drug to the target cell [13,14,20].

The chemical modification of HA can be used on three available functional components in the carboxylic, hydroxyl, and acetamido groups [21]. There are many modification methods for HA crosslinking or conjugation due to HA solubility [22]. The carboxylic group of HA could be exploited for controlled chemical modification with different hydrazides to obtain polymers that could be used for many biomedical applications, including developing prodrugs [23]. Another approach is activation of the hydroxyl group of paclitaxel with carbodiimide for conjugation with 4-bromobutyric acid to form ester-linked 4-bromobutyric-paclitaxel [2]. The commonly used chemotherapy drugs in the clinical field, paclitaxel (PTX) and doxorubicin (DOX), have also shown great results. PTX is one of the leading compounds and has tremendous potential as an anticancer compound. However, intravenous injection of PTX is difficult due to hydrophobicity and side effects. PTX conjugation with hydrophilic HA can overcome these limitations [24].

### 2.2. Micelles

**Figure 2:**
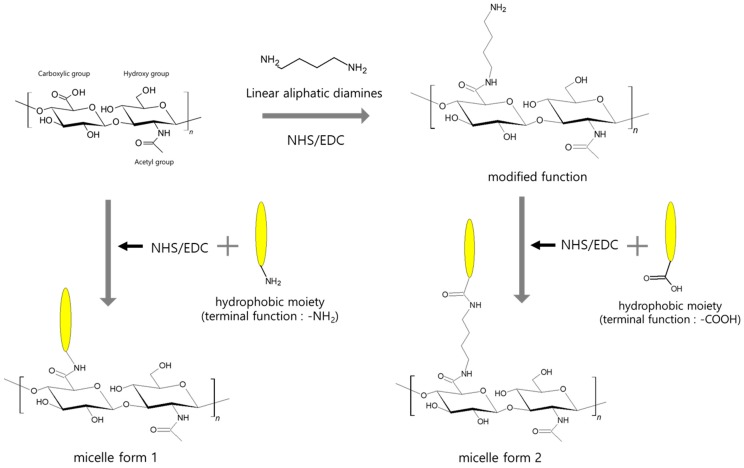
Chemical modification of hyaluronic acid for binding hydrophobic molecules to form micelles.

HA can form self-assembling micelles to generate amphiphilic nanocarriers. Micelles that are 20–80 nm in diameter are colloidal dispersions that constitute an amphiphilic molecule. Smaller size of micelles could limit the ability to administer large doses of chemotherapeutic agents to tumors [25]. Besides, the hydrophilicity of HA micelles increased circulation times of drugs in vivo, so they accumulated within cancer cells [26,27]. HA micelles can efficiently carry hydrophobic drugs to target cancer cells, thus enhancing the bioavailability and half-life of the drugs [12]. The solubilization of hydrophobic drugs is an important factor to decrease the risk of drug aggregation and embolism formation during intravenous injection, as shown in Figure 2 [28]. HA micelles might have great tumor tissue penetration compared with the large size of liposomes [25].

### 2.3. Polymersome

HA has been used as a surface ligand to exploit CD44-targeted liposomal anticancer therapies. Liposomes have been widely used as a nanodelivery system [29]. Polymersomes, that are a self-assembly of amphiphilic diblock copolymers in an aqueous environment, are similar to liposomes that form synthetic bilayer vesicles. Polymersome is able to encapsulate water-soluble drugs as well as lipophilic drugs [30,31]. Biocompatible polymersomes do not respond with blood components, and do not affect nontarget tissues. The polyethylene glycols (PEG) are the most commonly used biopolymer to produce biocompatible polymersomes. Polymersomes offer significant advantages over other bilayer vesicles. Compared to liposomes, the advantages of polymersomes are high membrane stability and low membrane permeability, resulting from varying the block lengths [30,31,32].

DOX-loaded polyglutamate-HA polymersomes were introduced. In this system, the HA-based polymersomes have advantages in both solubility and the targeting ability of CD44 receptors expressing cancer cells [33,34].

### 2.4. Hydrogels

**Figure 3:**
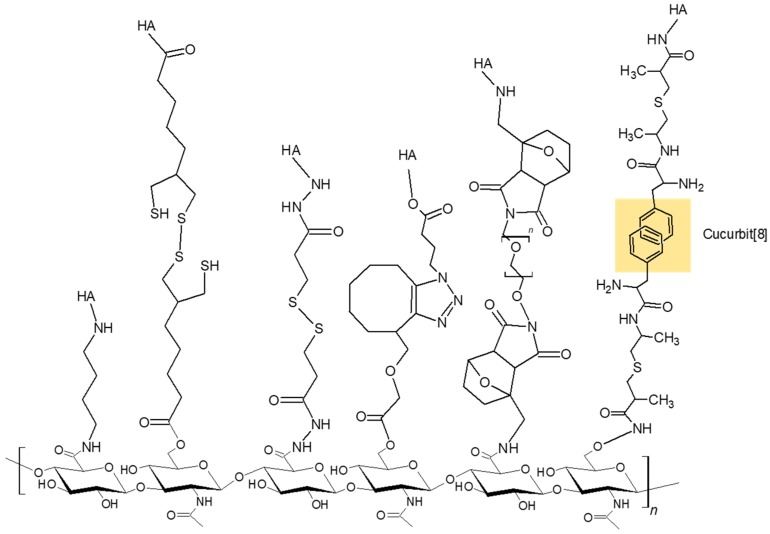
Chemical modification of hyaluronic acid to form hydrogels.

HA modification using click chemistry or supermolar assemblies is widely used to produce the covalent or physical hydrogels, as shown in Figure 3 [35,36]. Covalent crosslinking of HA is important to enhance the stability and function. Conventional HA-based hydrogels are macroscopic networks consisting of randomly interconnected HA chains at the crosslinking points established by covalent bonds [37].

Micro- or nano-sized hydrogels possess the possession of a high water content and desirable mechanical properties and biocompatibility. Drug molecules encapsulated without any covalent linkages or other specific interactions are released rapidly due to the relatively huge pore size [37]. The hydrophilic hydrogels are a good DDS due to tunable viscoelasticity, low cytotoxicity, further bioconjugation, as well as the prevention of enzymatic degradation. The hydrophilic internal space prevents aggregation of the drug payload [38].

### 2.5. Inorganic NPs

Inorganic matrices, such as iron oxide NPs, gold NPs, silica NPs, graphene oxide (GO), and quantum dots (QD), have been researched in cancer imaging, drug delivery, and various theranostics [39]. However, inorganic NPs have potential cytotoxicity and a lack of cell-specific function. Therefore, combined use of various biopolymers, especially polysaccharides, decreases the inorganic NPs’ toxicity and enhances biocompatibility and stability [4]. Nowadays, inorganic NPs by conjugation with HA have several new features and enhance their target ability.

Lee et al. [40] showed superparamagnetic iron oxide NPs coated with near-infrared (NIR) fluorescence dye (Cy5.5)-labeled HA (Cy5.5-HA). These NPs have potential as a dual imaging system for magnetic resonance and enzyme-sensitive optical imaging.

Recently, mesoporous silica nanoparticles (MSNs) have been introduced as useful DDS. MSNs have recently been important parts of NP systems due to their easy synthesis, large loading capacity, turnable pore size, good chemical stability, and good biocompatibility [41]. Conjugation of HA onto the surface of MSNs efficiently prevented their aggregation in physiological fluids and enabled efficient MSN uptake by CD44-overexpressing cells, while cells with low CD44 expression, such as MCF-7 and L929 cells, showed low uptake [42].

Biomedical scientists have had great interest in GO and its derivatives, such as single-layered carbon materials. GO and its derivative’s 2D structure provides a huge surface area, so it easily conjugates with biomolecules. GO has additional ability, such as optical properties for biomedical imaging and photothermal therapy (PTT) of cancer [43]. HA-GO-DOX nanomaterials are synthesized as a targeted and pH-responsive DDS for controlling the release of DOX for cancer therapy. This NP compared with free DOX demonstrated enhanced cancer inhibition rate in hepatoma mice models [44].

QDs are semiconductor nanocrystals. QDs have attracted attention in the biomedical field due to their bioimaging features. QDs have special light-emitting properties by tuning their size and composition [45]. However, their intrinsic toxicity limits possible biological applications.

## 3. Degradation of HA

HA is catalyzed by two main processes: enzymatic and non-enzymatic. Enzymatic degradation of HA-based nanomaterials is catalyzed by hyaluronidase in biological environments. HA is primarily degraded through enzymatic processes that hydrolyze the β-1,4-glycosidic bonds. This process occurs through the action of three enzymes. Hyaluronidase cleaves high molecular weight HA into smaller oligosaccharides. Next, β-d-glucuronidase and β-M-acetyl-hexosaminidase further degrade the oligosaccharide fragments [46,47]. These enzymes broadly distribute in the body tissues and operate in wide range of pH [48]. Catalase inhibits the degradation of HA macromolecules in a dose-dependent manner, which possibly makes H_2_O_2_ in the reaction system [49].

A non-enzymatic degradation process for HA has been reported in the presence of reactive oxygen species [50]. The resulting products considerably differ from the parent HA, while the products originating from the enzymatic degradation constitute parent HA-chains of smaller molecular weights [51]. Following an intravenous injection, HA, which has a half-life of 3–6 min, is rapidly removed from the blood circulation. Using labeled HA, it was determined that the liver and spleen are the main pathways for HA removal, with as much as 88% of injected HA absorbed by the liver. Thus, the primary and rapid clearance pathway for HA is through the liver [52]. Renal clearance of HA occurs as only 1% of the normal daily turnover of HA [47].

## 4. Cancer Therapy via HA-Based Nanomaterials

Most cancer patients undergo a combination therapies including surgery, chemotherapy, and/or radiation treatment. Recently, targeted chemotherapy, gene delivery, and immunotherapy strategies are being tested in translational and clinical research. HA-based nanomaterials are effective in many cancer treatments. Several groups have reported attractive outcomes in vitro and preclinical studies about targeting drug-loaded NP with HA polymer [53]. Conjugating drugs with HA enhances the nanocarrier affinity and it enhances great tumor cell targeting ability via the HA–CD44-mediated endocytosis [19].

### 4.1. Chemotherapeutic Agents

Anticancer chemotherapeutic agents have commonly toxic side effects and this limits the patient drug dose [53]. The use of HA-based nanomaterials is a commonly used DDS for chemotherapeutic agents. Nanomaterials enhance the overall pharmacological properties of widely used hydrophobic anticancer drugs, such as PTX, DOX, and irinotecan (IRT), as shown in Table 1.

Covalent conjugation with HA could produce the selective delivery of PTX to tumor cells. Several chemical strategies produce various HA-PTX nanomaterials with different chemical linkages and this nanomaterial has various performances [22]. HA solubilization method in a single organic phase is a chemical conjugation process of HA and PTX (HA-PTX). HA-PTX conjugate showed a greater inhibition effect on CD44-overexpressing cancer cells than that for normal cells [54]. Cai et al. [55] showed HA-DOX nanoconjugates. These nanoconjugates had a lasting release feature in the rodent breast cancer models and reduced cardiac toxicity and showed minimal toxic effects in healthy tissue. Finally, the conjugates inhibited breast cancer progression in rodent breast cancer models and showed good anticancer effect.

**Figure 4:**
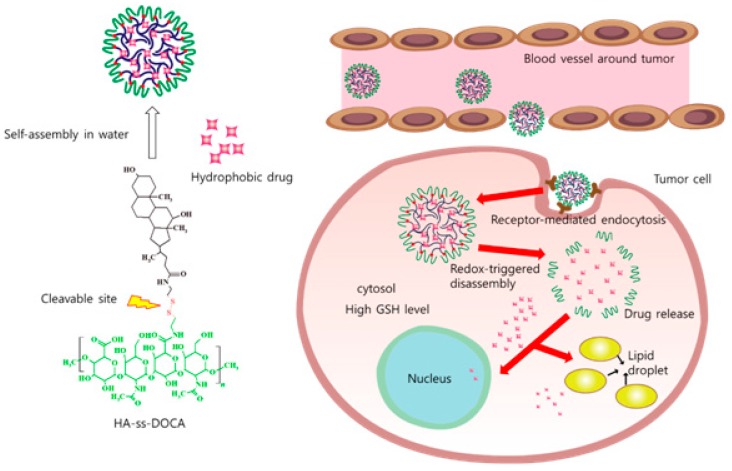
Illustration of redox-sensitive HA-ss-DOCA micelles (Modified from Reference [48]).

Several reports showed that the micelles have great drug-loading capacities for hydrophobic chemotherapeutic agents [56,57]. The PTX-loaded micelle was successfully synthesized based on redox-sensitive HA-deoxycholic acid (HA-ss-DOCA) conjugates, as shown in Figure 4. These micelles had effective intracellular anticancer drug delivery systems of lipophilic drugs and could significantly increase their accumulation in the tumor site [57]. Our group [56] synthesized PTX-loaded HA micelles targeting CD44-overexpressing cancer cells (SCC7 cells). PTX-loaded micelle labeled with NIR dyes were injected into SCC7 cancer mice model for theranostic test. PTX-loaded HA micelles have potential as a specific anticancer treatment effect for SCC5 cancer cells. Choi et al. [58] synthesized PEG-conjugated HA micelle (PEG-HA) and encapsulated the IRT into the hydrophobic cores of the PEG-HA micelle. Due to their remarkable tumor-targeting ability, IRT-loaded micelle showed great antitumor activity while reducing undesirable side effects.

Paliwal et al. [59] reported the synthesis of HA-based pH-sensitive (pH-HA) polymersomes. pH-HA polymersomes had characteristics of pH dependent DOX release that was significantly increased at pH of ~5 compared with a physiological pH ~7.4. The antitumor efficacy of these polymersomes was proven in CD44 receptor expressing MCF-7 cells. pH-sensitive HA-based polymersomes were significantly more effective than the non-targeted polymersomes in MCF-7 cells.

PTX-loaded HA hydrogel was tested as a carrier of PTX for the intraperitoneal drug delivery to the intraperitoneal ovarian cancer model. When administered to tumor-bearing nude mice, PTX hydrogel showed relatively good retention in the intraperitoneal cavity of mice model rather than PTX gel after intravenous injection. However, PTX hydrogel did not reduce the tumor volume more than PTX, presumably due to the limited dissolution of PTX [60].

Li et al. [61] designed and synthesized novel MSN NPs chemically modified with HA to develop a tumor-targeting DDS for treating MCF-7 breast cancer. They demonstrated that PTX-loaded HA-MSN NPs increased the accumulation of PTX in breast cancer cells via CD44-mediated endocytosis and showed great anticancer effect in vitro. The HA-MSN NPs possessed a preferable anticancer ability and exhibited not only minimal toxic side effects but a strong cancer suppression potential of PTX as well.

Another researcher reported HA-DOX NPs conjugated by an acid-labile hydrazone linkage. Due to specific biological cognition of HA, these NPs have the feature of good water solubility, and specific accumulation in human hepatoma model (HepG2 cells). In addition, the cumulative release of DOX from these NPs was significantly increased at acidic pH of 5.0–6.0 compared with its release at the physiological pH of 7.4. The HA-DOX conjugate might be effective chemotherapy against hepatoma [62].

### 4.2. Gene Delivery

Anticancer therapy using an RNA interference mechanism has good potential for sequence-specific silencing of target genes. Because naked small interfering RNA (siRNA) are easily degraded, viral-mediated siRNA delivery has been examined for gene delivery systems. Compared with viral gene delivery vectors that have the drawbacks of potential toxicity and immunogenicity, HA nanodelivery systems are an attractive alternative carrier for siRNAs. siRNA encapsulated HA nanocarriers showed specific tumor uptake and gene knockdown in vivo in solid and metastatic tumors [63]. The HA-based nanomaterials may be used in siRNA delivery systems for treatment of CD44 expressing cancers, as shown in Table 2.

HA conjugated to polyethyleneimine (PEI), when mixed with siRNAs, formed a self-assembling amphiphilic system for targeted siRNA delivery. Many HA derivatives stably encapsulate/complex siRNAs and form self-assembled nanocarriers. HA nanomaterials loaded with Cy3-labeled siRNA showed great inhibition of the receptor-mediated uptake, confirming target specificity. siRNA encapsulated in HA-PEI nanocarriers showed dose-dependent and target-specific gene knockdown in CD 44 expressing lung cancer cells [64]. Jiang et al. [65] also demonstrated that the inhibition effect of an siRNA/PEI-HA complex to mouse melanoma cells was lower than that of an siRNA/PEI complex.

siRNA-HA conjugate might be engulfed by HCT-116 cells having a CD44 positive receptor. Green fluorescence protein (GFP) siRNA encapsulated within the HA carriers could modify the release rates of siRNA depending on glutathion concentration. The siRNA-HA conjugate can be used as a new therapeutic delivery platform for siRNA [66].

Transferrin (Tf) and HA ligand-decorated, plasmid-enhanced GFP-loaded (Tf/HA-pDNA) polymersome was synthesized. The gene transfection efficiency of polymersome was evaluated in A549 lung adenocarcinoma mice models. HA-based polymersome enhanced the cell-targeting ability by CD44 receptor-mediated endocytosis. HA polymersome showed low cytotoxicity and an excellent gene delivery effect, and might be a promising tool as effective gene therapy for lung cancer [67].

Lee et al. [68] synthesized calcium phosphate (CAP) NPs conjugated of 3,4-dihydroxy-l-phenylalanine (dopa) and HA for a drug delivery carrier of siRNA. These NPs effectively protected siRNA from enzymatic degradation. This NP system showed high intratumoral concentration of siRNA and great target gene silencing in solid tumors. This siRNA delivery system might be effective for targeted cancer therapy.

### 4.3. Immunotherapy

Immunotherapy has become an appealing therapeutic option and a major component in successful cancer treatment [69]. Immunostimulatory effect of biomolecules encapsulated within or conjugated with nanomaterials are shown to elicit enhanced T- and B-cell responses as compared to the biomolecules delivered in a soluble form. HA-based nanomaterials in cancer immunotherapy is recently one of the most appealing fields in cancer therapy, as shown in Table 3. Zamboni et al. [70] demonstrated that HA has the capability of a protection effect from the immune response, and an immune modulation effect because of its intrinsic anti-inflammatory and anti-immunogenic functions. HA has the potential as a cancer therapy system of immunotherapy.

Conjugates consisting of HA and ovalbumin (OVA) as a foreign antigen was tested. When nanomaterials treated with CD44 expressing murine cervical cancer cells, it effectively internalized and enabled display of the antigenic OVA peptide on the cell surface. HA-OVA effectively accumulated into the mouse tumor model after its systemic intravenous injection [71]. Another group synthesized matrix metalloproteinase 9 (MMP9)-responsive nanomaterials consisting of PEGylated HA and OVA peptide. This nanosystem in the presence of MMP-9 entered to the murine cervical cancer cell line by receptor-mediated endocytosis. The nanomaterials systemically administered to tumor-bearing mice with endogenous OVA-specific cytotoxic T cells demonstrated the inhibition of cancer growth [72].

Xu et al. [69] synthesized lipid-calcium-phosphate (LCP) and liposome-protamine-HA (LPH) polymersomes. Effective immunoresponses against self/cancer antigens and great therapeutic results against advanced cancer are major challenges in cancer immunotherapy. LCP vaccine nanomaterials delivered both a tumor antigen and adjuvant to dendritic cells and promoted a systemic immune response regardless of the stage of host cancer. LPH NPs loaded siRNA made a 50% knockdown of TGF-β in the late-stage tumor microenvironment. Combination of LCP and LPH nanomaterials might be a powerful platform for cancer immunotherapy compared with vaccine treatment alone.

Immunotherapy of interferon-alpha (IFN-α) is used for treatment for metastatic renal cell carcinoma (RCC). The synergistic effect of IFN-α-incorporated HA hydrogels plus sorafenib in a mouse model is reported. HA hydrogels most effectively inhibited tumor growth in nude mice having human RCC xenografts. In addition, these nanomaterials suppressed the proliferation of RCC cells by inducing apoptosis and inhibiting angiogenesis [73]. Shin et al. [74] synthesized HA-based combination vaccine adjuvants including hydrophobic monophosphoryl lipid, QS21, and imiquimod (R837). The immunostimulatory effects of HA-based vaccine adjuvants were effective in the restriction of tumor cell proliferation in a mouse lymphoma expressing OVA mouse model. This nanomaterial combined with vaccine adjuvants may be used as a potent cancer therapeutic vaccine.

### 4.4. Combination Therapy

Combination cancer therapies combining chemotherapy with gene, immunotherapy photodynamic (PDT), or photothermal therapy (PTT) is a promising new method for cancer treatment. To decrease the toxic effect on normal cells, multifunctional NPs encapsulating multiple chemotherapeutic drugs and/or gene therapeutic or immunotherapeutic agents are used to simultaneously deliver to increase the killing effects of cancer cells to inhibit different metabolic pathways [75]. As HA has several functional parts that replace, there is potential for the conjugation of more than one therapeutic agent to HA, and it may make a combination therapy, as shown in Table 4.

One report showed the combination of immunotherapy and chemotherapy to treat breast cancer using a dual pH-responsive nanomaterial system. Conjugates based on poly(l-histidine) and HA were designed for co-loading R848 and DOX and showed great tumor targeting, and significantly suppressed the 4T1 tumor growth by regulation of immunity and killing effect on tumor cells [76].

The combination therapy of chemotherapy and PDT is increased the therapeutic efficacy in treating cancer. HA-ceramide micelle encapsulating both a photosensitizer and anticancer drug efficiently enhanced PDT effect in a lung cancer model. Developed micelles are introduced as a good DDS in PDT [77].

Nanoplatform self-assembled from HA-PTX prodrug and marimastat (MATT)-loaded thermosensitive polymersome is synthesized for the dual targeting of the cancer cells and TME. This polymersome rapidly released their payloads into the extracellular environment, and the released HA-PTX entered 4T1 cells through the CD44 receptor-mediated endocytosis. This nanomaterial can be self-assembled into a smart polymersome and are effective treatment nanocarriers. The co-delivery of MATT and HA-PTX is a good therapeutic option of metastatic cancer [78].

Deng et al. [79] synthesized miR-34a, a potent endogenous breast cancer tumor-suppressive molecule, co-encapsulated with DOX into HA-chitosan (CS) hydrogels for targeting breast cancer cells. HA-CS NPs co-loaded miR-34a and DOX could have a synergistic anticancer effect by suppressing the oncogene and enhancing the antitumor effects of DOX on cancer cells. Combined chemotherapy and PTT provides new therapeutic drug delivery carriers for synergistic cancer therapy.

Combination of light-responsive GO, DOX, and pH-sensitive disulfide-bond linked HA form a nano-sized hydrogel. The nanogel showed excellent photoluminescence properties and good stability in buffer and serum solutions. The GO-DOX-HA hydrogel might be a very effective DDS with inhibition of human lung cancer cells and a decreased toxic effect on healthy cells [80].

Wang et al. [81] reported a multi-stimuli responsive nanoplatform based on DOX loaded gold (Au) nanocages of HA for targeted intracellular drug release. DOX-AuNCs-HA could specifically target cancer cells via CD44 endocytosis. DOX-AuNCs-HA could release encapsulated DOX and induce a higher therapeutic efficacy upon NIR irradiation and reduce the toxic effects.

## 5. Summary and Prospective Outlook

HA-based nanomaterials can target and enter cells more efficiently through the HA receptor-mediated endocytosis pathway. HA provides a simple and attractive approach for the active targeting of tumor cells with minimal toxicity. HA nanomaterials are attractive systems for the effective delivery of antitumor agents. Much research has demonstrated the ability of HA to target CD44-overexpressing cancer cells. Interestingly, HA can easily be chemically modified and used as a target-specific DDS or in various carrier systems for cancer therapy.

The advancement of nanomedicine has offered new and promising solutions and insights for the prevention and theranostic option of cancer. HA-based nanomaterials might give new opportunities for the widespread use of biomedical applications. As research in HA nanomaterials progresses, we expect more innovative strategies for expanding their biomedical applications. HA-based nanomaterials show great promise for future biomedical applications in cancer therapy.

## Figures and Tables

**Table 1:** Summary of recent principal research for chemotherapy using hyaluronic acid (HA)-based nanomaterials.

Formulation	Component	Status	Indication	Ref.
**Drug conjugation**	Paclitaxel-HA	In vitro	HCT-116 MCF-7	[54]
	Doxorubicin-HA	In vivo	MDA-MB-468LN (human breast cancer)	[55]
**Micelle**	5B-cholanic acid-HA, paclitaxel	In vivo	SCC7 (squamous cell carcinoma)	[56]
	Deoxycholic acid-HA, paclitaxel	In vivo	MDA-MB-231 (breast adenocarcinoma)	[57]
	5B-cholanic acid-HA-PEG, irinotecan	In vivo	HT29 (human colorectal adenocarcinoma)	[58]
**Polymersome**	DSPE-PEG-HA, doxorubicin	In vivo	MCF7 (human breast adenocarcinoma)	[59]
**Hydrogel**	paclitaxel	In vivo	SKOV-3 (human ovarian cancer)	[60]
**Nanoparticle (surface modification)**	Si nanoparticle, paclitaxel	In vivo	MCF-7 (human breast adenocarcinoma)	[61]
	SPION-HA, doxorubicin	In vivo	HepG2 (hepatocellular carcinoma)	[62]

**Table 2:** Summary of recent principal research for gene therapy using HA-based nanomaterials.

Formulation	Component	Status	Indication	Ref.
**Complex (electric interaction)**	Polyethyleneimine-HA, siRNA	In vivo	MDA-MB 468, A549, B16F10 (CD44 expressed cancer)	[64]
	Polyethyleneimine-HA, siRNA	In vitro	B16F1 (murine melanoma), HEK-293 (human embryonic kidney)	[65]
**Gene conjugation**	siRNA-HA	In vitro	HCT-116 (human colon carcinoma)	[66]
**Polymersome**	DSPE-PEG-HA, pDNA	In vitro	A549 (human lung adenocarcinoma)	[67]
**Nanoparticle (surface modification)**	Cancium phosphate-HA, siRNA	In vivo	HT29 (human colorectal adenocarcinoma)	[68]

**Table 3:** Summary of recent principal research for immunotherapy using HA-based nanomaterials.

Formulation	Component	Status	Indication	Ref.
**Drug (direct) conjugation**	HA-ovalbumin	In vivo	TC-1 (murine cervical cancer)	[71]
**Micelle**	PEG-pep-HA, ovalbumin	In vivo	TC-1 (murine cervical cancer)	[72]
**Polymersome**	DSPE-PEG-HA, siRNA (for TGF-β)	In vivo	B16F10 (melanoma)	[69]
**Hydrogel complex**	HA-tyramine, IFN-α, sorafenib	In vivo	ACHN (human renal adenocarcinoma)	[73]
	Monophosphoryl lipid, QS21, R837 + HA	In vivo	EG7-OVA tumor (mouse lymphocyte)	[74]

**Table 4:** Summary of recent principal research for combination therapy using HA-based nanomaterials.

Formulation	Component	Status	Indication	Ref.
**Drug conjugation**	R848 (immuno), HA-doxorubicin (chemo)	In vivo	4T1 (mammary carcinoma)	[76]
**Micelle**	Hypocrellin B (PDT), paclitaxel (chemo), HA-ceramide	In vivo	A549 (human lung adenocarcinoma)	[77]
**Polymersome**	Marimastat (TME), HA-paclitaxel (chemo)	In vivo	4T1 (mammary carcinoma)	[78]
**Hydrogel**	Doxorubicin (chemo), mRNA (gene), HA-chitosan	In vivo	MDA-MB-231 (human breast cancer)	[79]
	Graphene (PTT), doxorubicin (chemo), HA-disulfide	In vivo	A549 (human lung adenocarcinoma)	[80]
**Nanoparticle**	Gold nanoparticle (PTT), doxorubicin (chemo), HA-dopamine	In vivo	MDA-MB-231 (human breast cancer)	[81]

## References

[1] Wickens J.M., Alsaab H.O., Kesharwani P., Bhise K., Amin M.C.I.M., Tekade R.K., Gupta U., Iyer A.K. (2017). Recent advances in hyluronic acid-decorated nanocarriers for targeted cancer therapy. Drug Discov. Today.

[2] Mattheolabakis G., Milane L., Singh A., Amiji M.M. (2015). Hyaluronic acid targeting of CD44 for cancer therapy: From receptor biology to nanomedicine. J. Drug Target..

[3] Lapcik L., Lapcik L., De Smedt S., Demeester J., Chabrecek P. (1998). Hyaluronan: Preparation, structure, properties, and applications. Chem. Rev..

[4] Cai Z.X., Zhang H.B., Wei Y., Gong F.S. (2017). Hyaluronan-Inorganic Nanohybrid Materials for Biomedical Applications. Biomacromolecules.

[5] Zhang M.Z., Xu C.L., Wen L.Q., Han M.K., Xiao B., Zhou J., Zhang Y., Zhang Z., Viennois E., Merlin D. (2016). A Hyaluronidase-Responsive Nanoparticle-Based Drug Delivery System for Targeting Colon Cancer Cells. Cancer Res..

[6] Dosio F., Arpicco S., Stella B., Fattal E. (2016). Hyaluronic acid for anticancer drug and nucleic acid delivery. Adv. Drug Deliv. Rev..

[7] Prabhakar U., Maeda H., Jain R.K., Sevick-Muraca E.M., Zamboni W., Farokhzad O.C., Barry S.T., Gabizon A., Grodzinski P., Blakey D.C. (2013). Challenges and Key Considerations of the Enhanced Permeability and Retention Effect for Nanomedicine Drug Delivery in Oncology. Cancer Res..

[8] Mattheolabakis G., Rigas B., Constantinides P.P. (2012). Nanodelivery strategies in cancer chemotherapy: Biological rationale and pharmaceutical perspectives. Nanomedicine.

[9] Lokeshwar V.B., Mirza S., Jordan A. (2014). Targeting Hyaluronic Acid Family for Cancer Chemoprevention and Therapy. Adv. Cancer Res..

[10] Zamboni F., Keays M., Hayes S., Albadarin A.B., Walker G.M., Kiely P.A., Collins M.N. (2017). Enhanced cell viability in hyaluronic acid coated poly(lactic-co-glycolic acid) porous scaffolds within microfluidic channels. Int. J. Pharm..

[11] Souness A., Zamboni F., Walker G.M., Collins M.N. (2018). Influence of scaffold design on 3D printed cell constructs. J. Biomed. Mater. Res. B Appl. Biomater..

[12] Choi K.Y., Saravanakumar G., Park J.H., Park K. (2012). Hyaluronic acid-based nanocarriers for intracellular targeting: Interfacial interactions with proteins in cancer. Colloids Surf. B.

[13] Luo Y., Prestwich G.D. (1999). Synthesis and selective cytotoxicity of a hyaluronic acid-antitumor bioconjugate. Bioconjug. Chem..

[14] Luo Y., Ziebell M.R., Prestwich G.D. (2000). A hyaluronic acid-taxol antitumor bioconjugate targeted to cancer cells. Biomacromolecules.

[15] Leonelli F., La Bella A., Francescangeli A., Joudioux R., Capodilupo A.L., Quagliariello M., Migneco L.M., Bettolo R.M., Crescenzi V., De Luca G. (2005). A new and simply available class of hydrosoluble bioconjugates by coupling paclitaxel to hyaluronic acid through a 4-hydroxybutanoic acid derived linker. Helv. Chim. Acta.

[16] Li H., Liu Y.C., Shu X.Z., Gray S.D., Prestwich G.D. (2004). Synthesis and biological evaluation of a cross-linked hyaluronan-mitomycin C hydrogel. Biomacromolecules.

[17] Montagner I.M., Merlo A., Zuccolotto G., Renier D., Campisi M., Pasut G., Zanovello P., Rosato A. (2014). Peritoneal tumor carcinomatosis: Pharmacological targeting with hyaluronan-based bioconjugates overcomes therapeutic indications of current drugs. PLoS ONE.

[18] Banzato A., Bobisse S., Rondina M., Renier D., Bettella F., Esposito G., Quintieri L., Meléndez-Alafort L., Mazzi U., Zanovello P. (2008). A paclitaxel-hyaluronan bioconjugate targeting ovarian cancer affords a potent in vivo therapeutic activity. Clin. Cancer Res..

[19] Jaracz S., Chen J., Kuznetsova L.V., Ojima L. (2005). Recent advances in tumor-targeting anticancer drug conjugates. Bioorg. Med. Chem..

[20] Luo Y., Bernshaw N.J., Lu Z.R., Kopecek J., Prestwich G.D. (2002). Targeted delivery of doxorubicin by HPMA copolymer-hyaluronan bioconjugates. Pharm. Res..

[21] Schante C.E., Zuber G., Herlin C., Vandamme T.F. (2011). Chemical modifications of hyaluronic acid for the synthesis of derivatives for a broad range of biomedical applications. Carbohydr. Polym..

[22] Mero A., Campisi M. (2014). Hyaluronic Acid Bioconjugates for the Delivery of Bioactive Molecules. Polymers.

[23] Prestwich G.D., Marecak D.M., Marecek J.F., Vercruysse K.P., Ziebell M.R. (1998). Controlled chemical modification of hyaluronic acid: Synthesis, applications, and biodegradation of hydrazide derivatives. J. Control. Release.

[24] Jordan M.A., Wilson L. (2004). Microtubules as a target for anticancer drugs. Nat. Rev. Cancer.

[25] Blanco E., Kessinger C.W., Sumer B.D., Gao J. (2009). Multifunctional Micellar Nanomedicine for Cancer Therapy. Exp. Biol. Med..

[26] Wang J., Mongayt D., Torchilin V.P. (2005). Polymeric micelles for delivery of poorly soluble drugs: Preparation and anticancer activity in vitro of paclitaxel incorporated into mixed micelles based on poly(ethylene glycol)-lipid conjugate and positively charged lipids. J. Drug Target..

[27] Husseini G.A., Pitt W.G. (2008). Micelles and nanoparticles for ultrasonic drug and gene delivery. Adv. Drug Deliv. Rev..

[28] Degim I.T., Celebi N. (2007). Controlled delivery of peptides and proteins. Curr. Pharm. Des..

[29] Khan D.R., Rezler E.M., Lauer-Fields J., Fields G.B. (2008). Effects of drug hydrophobicity on liposomal stability. Chem. Biol. Drug Des..

[30] Bermudez H., Brannan A.K., Hammer D.A., Bates F.S., Discher D.E. (2002). Molecular weight dependence of polymersome membrane structure, elasticity, and stability. Macromolecules.

[31] Discher B.M., Won Y.Y., Ege D.S., Lee J.C.M., Bates F.S., Discher D.E., Hammer D.A. (1999). Polymersomes: Tough vesicles made from diblock copolymers. Science.

[32] Kamat N.P., Lee M.H., Lee D., Hammer D.A. (2011). Micropipette aspiration of double emulsion-templated polymersomes. Soft Matter.

[33] Upadhyay K.K., Le Meins J.F., Misra A., Voisin P., Bouchaud V., Ibarboure E., Schatz C., Lecommandoux S. (2009). Biomimetic Doxorubicin Loaded Polymersomes from Hyaluronan-block-Poly(gamma-benzyl glutamate) Copolymers. Biomacromolecules.

[34] Upadhyay K.K., Bhatt A.N., Mishra A.K., Dwarakanath B.S., Jain S., Schatz C., Le Meins J.F., Farooque A., Chandraiah G., Jain A.K. (2010). The intracellular drug delivery and anti tumor activity of doxorubicin loaded poly(gamma-benzyl l-glutamate)-*b*-hyaluronan polymersomes. Biomaterials.

[35] Burdick J.A., Prestwich G.D. (2011). Hyaluronic Acid Hydrogels for Biomedical Applications. Adv. Mater..

[36] Highley C.B., Prestwich G.D., Burdick J.A. (2016). Recent advances in hyaluronic acid hydrogels for biomedical applications. Curr. Opin. Biotechnol..

[37] Xu X., Jha A.K., Harrington D.A., Farach-Carson M.C., Jia X.Q. (2012). Hyaluronic acid-based hydrogels: From a natural polysaccharide to complex networks. Soft Matter.

[38] Oh J.K., Drumright R., Siegwart D.J., Matyjaszewski K. (2008). The development of microgels/nanogels for drug delivery applications. Prog. Polym. Sci..

[39] Arpicco S., Milla P., Stella B., Dosio F. (2014). Hyaluronic Acid Conjugates as Vectors for the Active Targeting of Drugs, Genes and Nanocomposites in Cancer Treatment. Molecules.

[40] Lee D.E., Kim A.Y., Saravanakumar G., Koo H., Kwon I.C., Choi K., Park J.H., Kim K. (2011). Hyaluronidase-Sensitive SPIONs for MR/Optical Dual Imaging Nanoprobes. Macromol. Res..

[41] Lu J., Liong M., Li Z.X., Zink J.I., Tamanoi F. (2010). Biocompatibility, Biodistribution, and Drug-Delivery Efficiency of Mesoporous Silica Nanoparticles for Cancer Therapy in Animals. Small.

[42] Ma M., Chen H.R., Chen Y., Zhang K., Wang X., Cui X.Z., Shi J.L. (2012). Hyaluronic acid-conjugated mesoporous silica nanoparticles: Excellent colloidal dispersity in physiological fluids and targeting efficacy. J. Mater. Chem..

[43] Akhavan O., Ghaderi E., Aghayee S., Fereydooni Y., Talebi A. (2012). The use of a glucose-reduced graphene oxide suspension for photothermal cancer therapy. J. Mater. Chem..

[44] Song E.Q., Han W.Y., Li C., Cheng D., Li L.R., Liu L.C., Zhu G.Z., Song Y., Tan W.H. (2014). Hyaluronic Acid-Decorated Graphene Oxide Nanohybrids as Nanocarriers for Targeted and pH-Responsive Anticancer Drug Delivery. ACS Appl. Mater. Interfaces.

[45] Pellegrino T., Manna L., Kudera S., Liedl T., Koktysh D., Rogach A.L., Keller S., Rädler J., Natile G., Parak W.J. (2004). Hydrophobic nanocrystals coated with an amphiphilic polymer shell: A general route to water soluble nanocrystals. Nano Lett..

[46] Stern R., Jedrzejas M.J. (2006). Hyaluronidases: Their genomics, structures, and mechanisms of action. Chem. Rev..

[47] Necas J., Bartosikova L., Brauner P., Kolar J. (2008). Hyaluronic acid (hyaluronan): A review. Vet. Med..

[48] Volpi N., Schiller J., Stern R., Soltes L. (2009). Role, Metabolism, Chemical Modifications and Applications of Hyaluronan. Curr. Med. Chem..

[49] Valachova K., Topol’ska D., Mendichi R., Collins M.N., Sasinkova V., Soltes L. (2016). Hydrogen peroxide generation by the Weissberger biogenic oxidative system during hyaluronan degradation. Carbohydr. Polym..

[50] Valachova K., Banasova M., Topol’ska D., Sasinkova V., Juranek I., Collins M.N., Šoltés L. (2015). Influence of tiopronin, captopril and levamisole therapeutics on the oxidative degradation of hyaluronan. Carbohydr. Polym..

[51] Jahn M., Baynes J.W., Spiteller G. (1999). The reaction of hyaluronic acid and its monomers, glucuronic acid and *N*-acetylglucosamine, with reactive oxygen species. Carbohydr. Res..

[52] Fraser J.R., Laurent T.C., Pertoft H., Baxter E. (1981). Plasma clearance, tissue distribution and metabolism of hyaluronic acid injected intravenously in the rabbit. Biochem. J..

[53] Maeda H., Wu J., Sawa T., Matsumura Y., Hori K. (2000). Tumor vascular permeability and the EPR effect in macromolecular therapeutics: A review. J. Control. Release.

[54] Lee H., Lee K., Park T.G. (2008). Hyaluronic acid-paclitaxel conjugate micelles: Synthesis, characterization, and antitumor activity. Bioconjug. Chem..

[55] Cai S.A., Thati S., Bagby T.R., Diab H.M., Davies N.M., Cohen M.S., Forrest M.L. (2010). Localized doxorubicin chemotherapy with a biopolymeric nanocarrier improves survival and reduces toxicity in xenografts of human breast cancer. J. Control. Release.

[56] Thomas R.G., Moon M., Lee S., Jeong Y.Y. (2015). Paclitaxel loaded hyaluronic acid nanoparticles for targeted cancer therapy: In vitro and in vivo analysis. Int. J. Biol. Macromol..

[57] Li J., Huo M.R., Wang J., Zhou J.P., Mohammad J.M., Zhang Y.L., Zhu Q.N., Waddad A.Y., Zhang Q. (2012). Redox-sensitive micelles self-assembled from amphiphilic hyaluronic acid-deoxycholic acid conjugates for targeted intracellular delivery of paclitaxel. Biomaterials.

[58] Choi K.Y., Jeon E.J., Yoon H.Y., Lee B.S., Na J.H., Min K.H., Kim S.Y., Myung S.J., Lee S., Chen X. (2012). Theranostic nanoparticles based on PEGylated hyaluronic acid for the diagnosis, therapy and monitoring of colon cancer. Biomaterials.

[59] Paliwal S.R., Paliwal R., Agrawal G.P., Vyas S.P. (2016). Hyaluronic acid modified pH-sensitive liposomes for targeted intracellular delivery of doxorubicin. J. Liposome Res..

[60] Bajaj G., Kim M.R., Mohammed S.I., Yeo Y. (2012). Hyaluronic acid-based hydrogel for regional delivery of paclitaxel to intraperitoneal tumors. J. Control. Release.

[61] Li J.M., Yang X.J., Yang P., Gao F.N. (2017). Hyaluronic acid-conjugated silica nanoparticles for breast cancer therapy. Inorg. Nano-Met. Chem..

[62] Fu C.P., Yang R.M., Wang L., Li N.N., Qi M., Xu X.D., Wei X.H., Jiang X.Q., Zhang L.M. (2017). Surface functionalization of superparamagnetic nanoparticles by an acid-liable polysaccharidebased prodrug for combinatorial monitoring and chemotherapy of hepatocellular carcinoma. RSC Adv..

[63] Ganesh S., Iyer A.K., Gattacceca F., Morrissey D.V., Amiji M.M. (2013). In vivo biodistribution of siRNA and cisplatin administered using CD44-targeted hyaluronic acid nanoparticles. J. Control. Release.

[64] Ganesh S., Iyer A.K., Morrissey D.V., Amiji M.M. (2013). Hyaluronic acid based self-assembling nanosystems for CD44 target mediated siRNA delivery to solid tumors. Biomaterials.

[65] Jiang G., Park K., Kim J., Kim K.S., Oh E.J., Kang H., Han S.E., Oh Y.K., Park T.G., Kwang Hahn S. (2008). Hyaluronic acid-polyethyleneimine conjugate for target specific intracellular delivery of siRNA. Biopolymers.

[66] Lee H., Mok H., Lee S., Oh Y.K., Park T.G. (2007). Target-specific intracellular delivery of siRNA using degradable hyaluronic acid nanogels. J. Control. Release.

[67] Zhang B., Zhang Y.Y., Yu D.M. (2017). Lung cancer gene therapy: Transferrin and hyaluronic acid dual ligand-decorated novel lipid carriers for targeted gene delivery. Oncol. Rep..

[68] Lee M.S., Lee J.E., Byun E., Kim N.W., Lee K., Lee H., Sim S.J., Lee D.S., Jeong J.H. (2014). Target-specific delivery of siRNA by stabilized calcium phosphate nanoparticles using dopa-hyaluronic acid conjugate. J. Control. Release.

[69] Xu Z.H., Wang Y.H., Zhang L., Huang L. (2014). Nanoparticle-Delivered Transforming Growth Factor-beta siRNA Enhances Vaccination against Advanced Melanoma by Modifying Tumor Microenvironment. ACS Nano.

[70] Zamboni F., Vieira S., Reis R.L., Oliveira J.M., Collins M.N. (2018). The potential of hyaluronic acid in immunoprotection and immunomodulation: Chemistry, processing and function. Prog. Mater. Sci..

[71] Lee Y.H., Yoon H.Y., Shin J.M., Saravanakumar G., Noh K.H., Song K.H., Jeon J.H., Kim D.W., Lee K.M., Kim K. (2015). A polymeric conjugate foreignizing tumor cells for targeted immunotherapy in vivo. J. Control. Release.

[72] Shin J.M., Oh S.J., Kwon S., Deepagan V.G., Lee M., Song S.H., Lee H.J., Kim S., Song K.H., Kim T.W. (2017). A PEGylated hyaluronic acid conjugate for targeted cancer immunotherapy. J. Control. Release.

[73] Ueda K., Akiba J., Ogasawara S., Todoroki K., Nakayama M., Sumi A., Kusano H., Sanada S., Suekane S., Xu K. (2016). Growth inhibitory effect of an injectable hyaluronic acid-tyramine hydrogels incorporating human natural interferon-alpha and sorafenib on renal cell carcinoma cells. Acta Biomater..

[74] Shin W.J., Noh H.J., Noh Y.W., Kim S., Um S.H., Lim Y.T. (2017). Hyaluronic acid-supported combination of water insoluble immunostimulatory compounds for anti-cancer immunotherapy. Carbohydr. Polym..

[75] Jia F., Liu X.P., Li L.H., Mallapragada S., Narasimhan B., Wang Q. (2013). Multifunctional nanoparticles for targeted delivery of immune activating and cancer therapeutic agents. J. Control. Release.

[76] Liu Y.Y., Qiao L.N., Zhang S.P., Wan G.Y., Chen B.W., Zhou P., Zhang N., Wang Y. (2018). Dual pH-responsive multifunctional nanoparticles for targeted treatment of breast cancer by combining immunotherapy and chemotherapy. Acta Biomater..

[77] Chang J.E., Cho H.J., Yi E., Kim D.D., Jheon S. (2016). Hypocrellin B and paclitaxel-encapsulated hyaluronic acid-ceramide nanoparticles for targeted photodynamic therapy in lung cancer. J. Photochem. Photobiol. B.

[78] Lv Y., Xu C., Zhao X., Lin C., Yang X., Xin X., Zhang L., Qin C., Han X., Yang L. (2018). Nanoplatform Assembled from a CD44-Targeted Prodrug and Smart Liposomes for Dual Targeting of Tumor Microenvironment and Cancer Cells. ACS Nano.

[79] Deng X.W., Cao M.J., Zhang J.K., Hu K.L., Yin Z.X., Zhou Z.X., Xiao X., Yang Y., Sheng W., Wu Y. (2014). Hyaluronic acid-chitosan nanoparticles for co-delivery of M1R-34a and doxorubicin in therapy against triple negative breast cancer. Biomaterials.

[80] Khatun Z., Nurunnabi M., Nafiujjaman M., Reeck G.R., Khan H.A., Cho K.J., Lee Y.K. (2015). A hyaluronic acid nanogel for photo-chemo theranostics of lung cancer with simultaneous light-responsive controlled release of doxorubicin. Nanoscale.

[81] Wang Z.Z., Chen Z.W., Liu Z., Shi P., Dong K., Ju E.G., Ren J.S., Qu X.G. (2014). A multi-stimuli responsive gold nanocage-hyaluronic platform for targeted photothermal and chemotherapy. Biomaterials.

